# Urban-rural difference in the associations between living arrangements and the health-related quality of life (HRQOL) of the elderly in China—Evidence from Shaanxi province

**DOI:** 10.1371/journal.pone.0204118

**Published:** 2018-09-20

**Authors:** Zhiying Zhou, Zhongliang Zhou, Jianmin Gao, Sha Lai, Gang Chen

**Affiliations:** 1 School of Public Health, Health Science Center, Xi’an Jiaotong University, Xi’an, Shaanxi, People’s Republic of China; 2 School of Public Policy and Administration, Xi’an Jiaotong University, Xi’an, Shaanxi, People’s Republic of China; 3 Monash Business School, Monash University, Melbourne, Australia; Old Dominion University, UNITED STATES

## Abstract

**Background:**

So far limited evidence exist comparing the difference between urban and rural elder residents in relation to how living arrangements correlates to health-related quality of life(HRQOL) of the elderly.

**Objective:**

This study aims to compare the HRQOL of the elderly with four living arrangements: living with spouse only (LS), living alone (LA), living with a spouse and adult children(LSC) and the single elderly living with adult children (SLC) in urban and rural areas of China.

**Methods:**

The data were drawn from the 2013 wave of Chinese National Health Service Survey in Shaanxi Province, which included 11,729 elderly people. The Chinese version of the EQ-5D-3L questionnaire was used to measure the HRQOL. Tobit regression model and logistic regression models were employed to estimate the associations between living arrangements and the HRQOL of the elderly.

**Results:**

The EQ-5D utility scores of the urban elderly with four different living arrangements (LS, LA,LSC and SLC) were 0.9141, 0.8392, 0.8176 and 0.9080, which were almost all higher than their rural counterparts. After controlling other confounding variables, tobit regression estimates showed that the EQ-5D utility scores of the single elderly either living alone or living with adult children were lower than the elderly living with a spouse in urban areas. In rural areas only the single elderly living with adult children were more disadvantaged. Additionally the logistic regression results showed living-alone elderly had worse psychological health and the single elderly living with adult children had worse physical health.

**Conclusion:**

The findings suggest that the urban elderly have better HRQOL than the rural elderly and the elderly with different living arrangements in urban and rural area have different HRQOL. More attention should be given to the poor mental health of the elderly living alone and the worse physical health of the single elderly living with adult children.

## Introduction

China has the largest elderly population in the world [1]. According to the United Nation (UN) Population Ageing Report in 2017, there were 22.8897 million people aged 60 and above in China, which accounted for 16.2% of the total population and this proportion is projected to increase to more than one third of its total (35.1%) by 2050 [2]. With this rapid aging process, the health of the Chinese elderly needs to be given more concern. Although advances in medical technology, improvements in lifestyle, and socioeconomic development in China has brought some benefits (e.g. lengthening people’s life spans), the overall health of the elderly population is worsening [3]. In 2015, The UN proposed the 2030 Sustainable Development Goals (SDGs), including one specific goal for health: ensuring healthy lives and promote well-being for all at all ages [4]. The increasing percentage and deteriorated health of the elderly population will add more challenges for China to achieve this health goal. Healthy ageing has become a major issue for policy makers and researchers.

As early as 2000, living arrangements has been identified as one of the most pressing concerns of the aging population by the UN [5]. Numerous studies have shown that living arrangements of older adults are closely associated with their health [6–10]. As an important factor to affect health, living arrangements can reflect the patterns of social networks and social support to the elders [11]. Living arrangements are also closely tied to caregiving responsibilities [12]. It is generally believed that coresidential arrangements are better than solitary arrangements in protecting the health of older persons since the former are more conducive to social support exchanges, healthy lifestyles, and economies of scale [13]. It is also true for a Chinese traditional value set for coresidence doesn’t only meet the demand for both sides to mutually support each other but also conform to the Confucian principle of filial piety that adult children are expected to care for their elderly parents [14,15]. However, as the result of the accelerated pace of urbanization and social development, the centuries-old tradition of extended-family living arrangement for the aged has been declining in the recent decades [16]. The proportion of the elderly not living with children and the proportion of one-couple-only households among the elderly population has increased considerably [17]. Besides, unlike some developed countries where the social support system for elderly has been in place for years, China has yet to formulate a well-established support system for the elderly. It is of great significance to analyze the effects of different living arrangements of the Chinese elderly on their health, which can not only provide some suggestions for improving the health of the old population, but also can offer some policy reference for building a more reasonable elderly supporting system.

An increasing number of cross-sectional studies and a few longitudinal studies conducted in China have indicated that living arrangements have an effect on mortality [18, 13], functional disability [19], cognitive impairment [20], psychological health [21–23], and self-rated health [24] of the old-age adults. Health-Related Quality of Life **(**HRQOL), which was defined as an individual’s or a group's perceived physical and mental health over time by the Center for Disease Control and Prevention (CDC) recently[25], has also been investigated as an important overall health outcome for the elderly with different living arrangements [24,26]. There were a lot of HRQOL measures, such as the Euro Qol 5-dimension questionnaire (EQ-5D), SF-36 and 12-item Short Form Health Survey (SF-12). Among those measures, the EQ-5D is currently the most widely used preference-based HRQOL measure internationally [27], including for Chinese [28–30]. Sun et al. (2011) has investigated the HRQOL of the Chinese urban elderly with three different living arrangement by using the EQ-5D inventory [24] and Liang and Wu (2014) compared the EQ-5D and SF-12 in measuring the HRQOL of the empty-nest elderly in rural China[26]. However, those studies were limited by the lack of Chinese population preference weights when analyzing the association between the elderly’s HRQOL and living arrangements. This study fills in the gap by adopting the Chinese-specific EQ-5D tariff [31].

How the living arrangements of the Chinese elderly were categorized in empirical studies varied in the literature. Some studies made a more generalized classification of the elders’ living arrangements [32,33], whilst others have a more multiple differentiation of living arrangements [13,19,20,22]. For example, Chou and Chi (2000) compared the subjective well-being of Chinese elderly living alone and those living with others[32], Qiang et al. (2015) examined the effect of 10 Living arrangements [22]. The literature on whether living alone or living with adult children are more beneficial to the health outcomes of the elderly shows inconsistent findings. For example, Li et al.’s study (2009) shows that Chinese older people who live alone have better health than the older people who live with adult children [13]; however, Sun et al. (2011) found that “living alone” was a significant predictor of reporting problems on Mobility, Pain/Discomfort and Anxiety/Depression among Chinese urban elderly [24]. Thus in this study, based on the previous literature and the actual living habits of the Chinese elders, we compare the following four living arrangements: living alone, living with spouse only, living with spouse and adult children and single elderly living with adult children.

Furthermore, the majority of previous studies focused only on the health of the elderly with different living arrangements either in the urban or the rural area of China [23, 26, 34]. Considering the significant urban-rural socioeconomic disparity in China, it will be ideal to study the associations in both urban and rural areas using the same analystical framework to facilitate the comparison. This study serves this aim by using a representative household survey data from a province with a total number of 38.35 million residents in 2017 in China [35].

The overall aim of this study was to investiagte the association between four mutually exclusive living arrangements and HRQOL of the elders by a large-scale representative survey data. The following two research questions will be answered: 1) Are different living arrangements closely related with the HRQOL of the elderly in China? 2) How do these relationships differ in urban and rural area and what related policy suggestions will be implied?

## Methods

### Data and sample

The data used in this study was derived from the fifth Health Service Survey of Shaanxi Province conducted in September of 2013, which was part of China’s National Health Service Survey (NHSS). As a representative survey, NHSS is a cross-sectional survey organized by the Health Statistics and Information Center of China’s Ministry of Health every five years.

In order to acquire the representative samples of rural and urban residents from Shaanxi province, a four-stage stratified random sampling procedure was applied, which involved first selecting 32 districts (in urban) and counties (in rural), 158 streets (urban) and townships (rural) in the second selection, then 326 community (urban) and villages (rural) in third stage and final selection of 20,700 households in total within the urban and rural area of Shaanxi Province. As the objects of the current study were the people aged 60 or above, the actual sample were 11,725, including 4447 urban elder residents and 7278 rural elder residents. By using face-to-face interviews, the questionnaire with the information on respondents’ demographic and socioeconomic status, health status (measured by the EQ-5D), medical insurance status, exercise habits, and chronic disease information were completed by the trained investigators.

### Variables and measures

#### Living arrangements

The living arrangements of the elderly were used as the key independent variable in the regression analysis. As explained in the introduction, four types of living arrangements were categorised: living with spouse only (LS), those who live only with his/her spouse; living alone (LA), those who live by himself/herself; living with spouse and adult children (LSC), those who live with spouse and their adult children; the single elderly living with adult children (SLC), those elderly who live with their adult children after bereft of their spouses.

#### Other covariates

Other confounding characteristics which may have some influential effect on the HRQOL of the elderly were also included in the regression analysis, including the economic status (measured by using annual individual expenditure), gender, age, educational status, medical insurance (including the basic medical insurance provided by government and other medical insurance), residential region (including three area of Shaanxi province: Shaannan, Guanzhong and Shanbei, which does not only represent the geographic distribution, but also reflect different levels of economic development), physical exercise (from never to ≥6 times per week), medical examination and whether respondents suffering from chronic diseases.

#### Measurements of HRQOL

In the fifth NHSS, the Chinese version of the three-level EQ-5D (EQ-5D-3L) was included. The validity and reliability of the Chinese version of the EQ-5D-3L have been tested in China in previous studies [36–38]. EQ-5D consists of five dimensions: mobility, self-care, usual activities, pain/discomfort and anxiety/depression. There are three ordinal levels of responses for each dimension: no problem, a moderate problem, or an extreme problem (coded with 1, 2 and 3). Thus, if all five dimensions are coded with 1 (i.e. “11111”), it means the respondents have no problems with their HRQOL, whilst if all five dimensions are coded with 3, (i.e. “33333”), it indicates the worst HRQOL. After adding “unconsciousness” and “death” into the health states, there are a total of 245 unique health states can be defined in EQ-5D system. The EQ-5D-3L health profile was then scored using the Chinese specific tariff developed by Liu et al [31] to generate the EQ-5D utility score, which ranges from −0.1490 (for the worst health status) to 1 (for the full health).

### Data analysis

Descriptive statistics including mean and proportion were reported for the demographic and social economic characteristics of the study sample. 95% confidence interval (CI) was used to estimate the difference of the EQ-5D utility scores of the elders in the urban and rural China. Since the dependent variable EQ-5D utility score usually exhibits a ceiling effect (i.e. a large proportion of respondents had the full health of 1), instead of the traditional ordinary least squares estimator, the tobit regression model was used to estimate the associations between living arrangements and the HRQOL of the elders in the urban and rural area [39].

In order to examine the different aspects of the elder’s health, we performed another logistic regression models (since those five dimensions are not continues variables) to analyze the association between the living arrangements and different dimensions of HRQOL of the elderly in different area. As dependent variables, the three levels of responses for each dimension were generalized into two catogaries (no problem and a moderate/extreme problem) in the logistic models, in which “no problem” was labeled 1 and “a moderate/extreme problem” were labeled 0.

The independent variables in the tobit regression model and logistic regression models included the living arrangements and other covariates. Except for the annual individual expenditure, all other variables were taken as dummy variables.

### Ethical considerations

The design of the fifth NHSS was approved by the National Bureau of Statistics of China (NO. 2013(65)). This study has been reviewed and approved by the Ethics Committee of Health Science Center of Xi’an Jiaotong University (No. 2015–644) and the requirement for the informed consent had been waived. All the data used in this study had been fully anonymized before we accessed them.

## Results

### Description of the sample

In urban area, the majority of elderly were categorized as lived with spouse only (LS) or lived with spouse and adult children (LSC), which accounted for 43.56% and 33.87% respectively, followed by another two living arrangements: SLC (12.57%) and LA (10.10%). Compared with the urban area, the corresponding proportions were slightly different in rural area, where the first two highest proportions of living arrangements (LS and LSC) were both lower than in urban area, which were 37.79% and 32.61% respectively, and there were more single elderly living alone (11.59%) and living with adult children (18.00%). Within the overall sample of 11,729 elder people (4,447 in urban area and 7,282 in rural area), the female elderly accounted for 52.26% in urban area and 49.48% in rural area. Among all the respondents, more than half of elderly were aged between 60 to 69 (29.95% elderly aged 60–64 and 24.24% elderly aged 65–69 in urban area, and the percentage of the couterpart in rural area are 35.94% and 25.31%), and there were 9.71% elders in urban area and 8.64% elders in rural area aged 80 and above. The detailed descriptive information of other covariates are shown in Table 1.

**Table 1:** Basic descriptive statistics of the elder people in this study (%).

Variables	Urban	Rural	Variables	Urban	Rural
**Living arrangements**			**Basic medical insurances**		
LS	43.56	37.99	Uninsured	1.75	7.21
LA	10.10	11.59	Insured	98.25	92.79
LSC	33.78	32.61	**Other medical insurance**		
SLC	12.57	18.00	Uninsured	96.85	96.90
**Annual individual** **Expenditure (RMB)**	8164.09	5287.29	Insured	3.15	3.10
**Gender**			**Residential region**		
Female	52.26	49.48	Shannan	25.97	35.16
Male	47.74	50.52	Guanzhong	64.27	49.09
**Age (years)**			Shanbei	9.76	15.75
60–64	29.95	35.94	**Physical exercise**		
65–69	24.24	25.31	Never	52.58	85.44
70–74	20.76	17.78	2 or 3 times/week	6.44	3.56
75–79	15.34	12.33	3 to 5 times/week	8.07	3.23
80 and above	9.71	8.64	≥6 times/week	32.92	7.76
**Education status**			**Medical examination**		
Illiterate	22.67	44.37	No	37.45	45.17
Primary school	30.85	37.35	Yes	62.55	54.83
Junior middle school	27.30	15.02	**Chronic diseases**		
Senior middle school	13.65	3.08	Without	49.38	56.89
Junior college	3.53	0.15	With	50.62	43.11
University and above	2.00	0.03			

Basic medical insurances (BMI) refer to the Urban Employee Basic Medical Insurance (UEBMI), the Urban Resident Basic Medical Insurance (URBMI) and the New Rural Cooperative Medical Scheme (NRCMS) in China.

### Description of the EQ-5D dimensions

Table 2 shows the proportion of the elderly who reported having any problems in the five dimensions of EQ-5D in urban and rural area. More rural elderly reported having problems (either moderate or extreme) in any of the five dimensions than urban elderly. Compared with the mobility and self-care dimensions, the elderly in rural area suffered more moderate or extreme problems in dimensions of usual activity, pain/discomfort and anxiety/depression than the elderly in urban area.

**Table 2:** The proportion of the elders reporting having any problems in each dimensions of EQ-5D (%).

Dimensions	Urban			Rural		
	No problem	Moderate problem	Extreme problem	No problem	Moderate problem	Extreme problem
**Mobility**	81.41	16.58	2.00	79.03	19.19	1.77
**Self-care**	88.93	8.80	2.27	87.02	10.77	2.21
**Usual activity**	84.97	11.65	3.37	80.57	15.39	4.04
**Pain/discomfort**	71.47	26.17	2.36	65.85	31.69	2.46
**Anxiety/depression**	86.97	11.72	1.31	83.33	15.53	1.14

The estimated utility scores of EQ-5D for elderly with four living arrangements in urban and rural area was shown in Table 3. As can be seen from the mean and 95% CI of the overall EQ-5D utility score, most urban elderly had better HRQOL than the rural elderly except those who live alone. In urban area, the overall EQ-5D utility score of elderly with the four living arrangements were, from high to low, living with spouse only (0.9141), living with spouse and adult children (0.9080), living alone (0.8392) and the single elderly living with adult children (0.8176). The EQ-5D utility scores ranked almost the same in rural area, and the EQ-5D utility scores of LS, LSC, LA and SLC are 0.0652, 0.0652, 0.8427 and 0.7720 respectively.

**Table 3:** The utility scores of the EQ-5D for the urban and rural elderly.

Living arrangements	Urban		Rural	
	Utility scores	95%CI	Utility scores	95%CI
**LS**	0.9141	0.9066, 0.9216	0.8652	0.8564, 0.8739
**LA**	0.8392	0.8178, 0.8607	0.8427	0.8269, 0.8585
**SLA**	0.8176	0.7965, 0.8387	0.7720	0.7552, 0.7889
**LSA**	0.9080	0.8985, 0.9174	0.8652	0.8548, 0.8755

### Regression results

In Table 4, tobit regression estimates are reported on the association between living arrangements and HRQOL of the elderly in urban and rural area after controlling other covariates. The results indicated that the HRQOL of the single elderly either living alone or with adult children were worse than the elderly living with spouse in urban area, which were statistically significant. We found no difference between the elderly living with spouse and the elderly living with spouse and adult children in regard to the EQ-5D utility score. In rural area, compared with the elderly living with spouse, only the single elderly living with adult children had worse health (dy = -0.0207) and there was statistically significant. In addition, we found that almost all the covariates were significant factors which influenced the HRQOL of the elderly in China.

**Table 4:** Results of tobit regression model for the overall EQ-5D utility score.

Variables	Urban		Rural	
	dy/dx	SE	dy/dx	SE
**Living arrangements (LS**^**#**^**)**				
** LA**	-0.0280***	0.0087	-0.0039	0.0090
** SLC**	-0.0243***	0.0082	-0.0207**	0.0082
** LSC**	-0.0022	0.0061	0.0063	0.0067
**Annual individual expenditure (RMB)**	0.0081**	0.0037	0.0177***	0.0036
**Male (Female**^**#**^**)**	0.0148**	0.0055	0.0228***	0.0058
**Age (60–64**^**#**^**)**				
** 65–69**	-0.0258***	0.0075	-0.0345***	0.0074
** 70–74**	-0.0548***	0.0077	-0.0792***	0.0080
** 75–79**	-0.0736***	0.0085	-0.1114***	0.0091
** ≥80**	-0.1184***	0.0096	-0.1485***	0.0103
**Education status (Illiterate**^**#**^**)**				
** Primary school**	0.0249***	0.0071	0.0244***	0.0063
** Junior middle school**	0.0239***	0.0080	0.0476***	0.0092
** Senior middle school or above**	0.0346***	0.0094	0.0298*	0.0166
**Basic medical insurances (Uninsured**^**#**^**)**	0.0054	0.0199	-0.0589***	0.0115
**Other medical insurance (Uninsured**^**#**^**)**	0.0258	0.0160	0.0271*	0.0161
**Residential region (Shannan**^**#**^**)**				
** Guanzhong**	0.0256***	0.0061	-0.0048	0.0061
** Shanbei**	-0.0230**	0.0094	-0.0283***	0.0081
**Physical exercise (0 times physical exercise/week**^**#**^**)**				
** 1–2 times physical exercise/week**	0.0185*	0.0107	0.0332**	0.0153
** 3–5 times physical exercise/week**	0.0367***	0.0100	0.0292*	0.0157
** 6 or above times physical exercise /week**	0.0662***	0.0066	0.0197*	0.0103
**Received medical examination (No examination**^**#**^**)**	0.0349***	0.0054	0.0326***	0.0056
**Suffering from chronic disease (No chronic disease**^**#**^**)**	-0.0886***	0.0054	-0.1073***	0.0055
**LR**	923.04		1069.31	
**P**	<0.0001		<0.0001	

^**#**^ Reference group

*p<0.10

**p <0.05

***p <0.01

dy/dx is the partial effect in Tobit regression model; The three living arrangements: living alone (LA), single elderly living with adult children,(SLC), living with spouse and adult children (LSC).

In the five dimensions of EQ-5D, the mobility, self-care, activity and pain/discomfort are used to describe the physical health and the anxiety/depression is for measuring the mental health. Table 5 shows the logistic regression estimates of each EQ-5D dimension for the elderly in urban area. Compared with the elders living with spouse, the elderly living alone had worse health in the dimensions of mobility(OR = 0.7688), selfcare(OR = 0.6934), pain(OR = 0.5910) and anxiety(OR = 0.7202) and the single elderly living with adult children reported more problems in dimension of mobility(OR = 0.7300), self-care(OR = 0.5842) and activity(OR = 0.7009), which were statistically significant. There was no statistically significant difference between the urban elderly living with spouse and the elderly living with spouse and adult children in relation to all five dimensions of HRQOL. As Table 6 shows, the living-alone single elderly in rural area had more health problems in anxiety/depression than other three living-arrangement elderly (OR = 0.8185). Compared with the elderly living with spouse, the single elderly living with adult children had more health problems in mobility, self-care and activity(OR = 0.7863, 0.7655, 0.7646). There was also no significant difference in the health status of the five dimensions between the elderly living with spouse and the elderly living with spouse and adult children in rural area.

**Table 5:** Results of the logistic regression models for utility scores of EQ-5D five dimensions of the elderly in urban area.

	Mobility		Selfcare		Activity		Pain		Anxiety	
	OR	SE	OR	SE	OR	SE	OR	SE	OR	SE
**Living arrangements (LS**^**#**^**)**										
**LA**	0.7688*	0.1131	0.6934**	0.1234	0.7709	0.1235	0.5910***	0.0749	0.7202**	0.1157
**SLC**	0.7300**	0.0980	0.5842***	0.0933	0.7009**	0.1013	0.9025	0.1092	1.0162	0.1601
**LSC**	1.0058	0.1095	0.8617	0.1195	0.8767	0.1053	1.0577	0.0957	0.8990	0.1081
**Annual individual expenditure(RMB)**	1.1566**	0.0726	1.1085	0.0844	1.0867	0.0741	1.0743	0.0585	1.1193	0.0773
**Male (Female**^**#**^**)**	1.1335	0.1089	1.1691	0.1403	1.1627	0.1226	1.2004**	0.0970	1.1160	0.1199
**Age (60–64**^**#**^**)**										
**65–69**	0.7073**	0.0997	0.7069*	0.1293	0.6629***	0.1052	0.6857***	0.0750	0.8784	0.1285
**70–74**	0.4513***	0.0634	0.4471***	0.0807	0.3902***	0.0610	0.5104***	0.0578	0.6368***	0.0957
**75–79**	0.2462***	0.0355	0.2717***	0.0495	0.2547***	0.0410	0.5055***	0.0631	0.5696***	0.0925
**≥80**	0.1626***	0.0260	0.1313***	0.0252	0.1153***	0.0199	0.3755***	0.0534	0.4190***	0.0748
**Education status (Illiterate**^**#**^**)**										
**Primary school**	1.1896	0.1388	1.2589	0.1787	1.1911	0.1490	1.4454***	0.1477	1.3919***	0.1783
**Junior middle school**	1.3331**	0.1831	1.2128	0.2060	1.4647**	0.2219	1.4907***	0.1734	1.3814**	0.2095
**Senior middle school or above**	1.4877**	0.2439	1.1587	0.2350	1.4933**	0.2694	2.0612***	0.2885	1.9266***	0.3769
**Basic medical insurances (Uninsured**^**#**^**)**	1.1712	0.3984	1.2847	0.5348	0.8966	0.3553	1.2119	0.3444	0.6893	0.3347
**Other medical insurance (Uninsured**^**#**^**)**	1.0259	0.2848	1.5247	0.5828	0.9868	0.2987	1.0598	0.2434	1.7042	0.6059
**Residential region (Shannan**^**#**^**)**										
**Guanzhong**	0.9706	0.1037	1.1723	0.1497	1.0093	0.1167	1.5593***	0.1385	2.4697***	0.2738
**Shanbei**	0.5123***	0.0814	0.6377**	0.1237	0.5099***	0.0875	0.7311**	0.0993	1.2533	0.2124
**Physical exercise (0 times physical exercise/week**^**#**^**)**										
**1–2 times physical exercise/week**	1.3687*	0.2570	1.8300**	0.4490	1.5822**	0.3312	1.0157	0.1593	1.5482*	0.3481
**3–5 times physical exercise/week**	1.7264***	0.3050	2.0814***	0.4731	1.4670**	0.2689	1.2979*	0.1891	1.7184**	0.3650
**6 or above times physical exercise /week**	2.6811***	0.3150	4.6759***	0.7917	3.8024***	0.5293	1.8261***	0.1746	2.5337***	0.3557
**Received medical examination (No examination**^**#**^**)**	1.4610***	0.1347	1.7989***	0.2001	1.6397***	0.1635	1.3006***	0.1038	1.6620***	0.1688
**Suffering from chronic disease (No chronic disease**^**#**^**)**	0.3322***	0.0310	0.3095***	0.0366	0.3282***	0.0337	0.3252***	0.0254	0.3326***	0.0352
**LR**	631.03		570.97		659.80		597.72		453.59	
**P**	<0.0001		<0.0001		<0.0001		<0.0001		<0.0001	

^#^ Reference group

*p<0.10

**p <0.05

***p <0.0

**Table 6:** Results of the logistic regression models for utility scores of EQ-5D five dimensions of the elderly in rural area.

	Mobility		Selfcare		Activity		Pain		Anxiety	
	OR	SE	OR	SE	OR	SE	OR	SE	OR	SE
**Living arrangements (LS**^**#**^**)**										
**LA**	1.0160	0.1073	0.9003	0.1125	1.0315	0.1135	0.9159	0.0810	0.8185*	0.0884
**SLC**	0.7863***	0.0722	0.7655**	0.0832	0.7646***	0.0723	0.9060	0.0735	0.9418	0.0941
**LSC**	1.1166	0.0893	1.0765	0.1064	1.0305	0.0857	1.0704	0.0697	1.0064	0.0844
**Annual individual expenditure(RMB)**	1.1790***	0.0492	1.2373***	0.0615	1.2063***	0.0520	1.1541***	0.0413	1.1601***	0.0511
**Male (Female**^**#**^**)**	1.1954***	0.0808	1.2459***	0.1012	1.2384***	0.0868	1.2144***	0.0690	1.3283***	0.0955
**Age (60–64**^**#**^**)**										
**65–69**	0.5833***	0.0536	0.5282***	0.0630	0.5268***	0.0513	0.8284***	0.0596	0.8494*	0.0802
**70–74**	0.4013***	0.0382	0.3290***	0.0393	0.3480***	0.0347	0.5691***	0.0443	0.5921***	0.0579
**75–79**	0.2724***	0.0283	0.2400***	0.0306	0.2410***	0.0261	0.4507***	0.0402	0.5345***	0.0588
**≥80**	0.1653***	0.0189	0.1332***	0.0178	0.1408***	0.0166	0.4227***	0.0435	0.4323***	0.0527
**Education status (Illiterate**^**#**^**)**										
**Primary school**	1.3133***	0.0963	1.1655*	0.1029	1.3580***	0.1030	1.2458***	0.0766	1.2599***	0.0973
**Junior middle school**	1.3160**	0.1421	1.2365	0.1668	1.4658***	0.1696	1.5684***	0.1418	1.6174***	0.1998
**Senior middle school or above**	1.8696***	0.4126	1.4076	0.3712	1.4592*	0.3156	1.3276*	0.2143	1.1340	0.2403
**Basic medical insurances (Uninsured**^**#**^**)**	0.6784***	0.0912	0.8654	0.1317	0.7562**	0.1040	0.5182***	0.0608	0.7308**	0.1103
**Other medical insurance (Uninsured**^**#**^**)**	1.0984	0.2096	1.1402	0.2712	1.4182	0.3018	1.3419*	0.2142	2.0201***	0.4808
**Residential region (Shannan**^**#**^**)**										
**Guanzhong**	0.7844***	0.0568	0.7235***	0.0632	0.9364	0.0696	1.0156	0.0609	1.5351***	0.1144
**Shanbei**	0.5517***	0.0509	0.6131***	0.0682	0.6400***	0.0607	0.8113**	0.0647	1.2072*	0.1183
**Physical exercise (0 times physical exercise/week**^**#**^**)**										
**1–2 times physical exercise/week**	0.9300	0.1590	1.1843	0.2604	1.1367	0.2116	1.3494**	0.2054	1.4181*	0.2902
**3–5 times physical exercise/week**	1.2910	0.2522	1.6618*	0.4366	1.605**	0.3477	1.1333	0.1727	1.7734**	0.4047
**6 or above times physical exercise /week**	1.1336	0.1389	1.1706	0.1763	1.1929	0.1538	1.2298**	0.1248	1.2833*	0.1725
**Received medical examination (No examination**^**#**^**)**	1.4335***	0.0930	1.4979***	0.1155	1.4603***	0.0979	1.1090*	0.0611	1.2946***	0.0889
**Suffering from chronic disease (No chronic disease**^**#**^**)**	0.4247***	0.0268	0.4698***	0.0355	0.4089***	0.0268	0.4058***	0.0216	0.4203***	0.0282
**LR**	837.30		626.05		896.85		716.60		480.97	
**P**	<0.0001		<0.0001		<0.0001		<0.0001		<0.0001	

^#^ Reference group

*p<0.10

**p <0.05

***p <0.01.

## Discussion

This study compared the HRQOL of elder adults with different living arrangements in urban and rural areas by using a large-scale representative household survey data. The descriptive results indicateds that there were more single elderly either living alone or living with adult children in rural area than in urban area. This is consistent with the latest national population census data, which showed that there were more single elderly, either being never married or berert of his/her spouse in rural areas of China [40]. Besides, more rural elderly reported having problems (either moderate or extreme) in any of the five dimensions than urban elderly, which was consistent with the previous studies on urban-rural health disparities among Chinese residents [41–45]. In China, it is commonly known that the urban residents enjoy more socioeconomic advantages, such as better education, higher employment, and better access to health care facilities. Health status closely follows a socioeconomic gradient, the lower an individual’s socioeconomic status, the worse their health [45]. Besides, the rural elderly is simultaneously experiencing “being left behind”, due to the vast rural-to-urban migration of the younger generation, which greatly weakened the tradition that the elderly relying on their adult children at their old age and would also cause great impact on their well-being. Dong and Simon’s research (2010) also indicated that the rural elderly reported significant lower overall health status, lower quality of life and worse change in recent health compared with the urban elderly [44].

By comparing the estimated utility scores of the EQ-5D among the elderly with four living arrangements in urban and rural area, we can see that both in urban and rural area, the single elderly either living alone or living with adult children were more disadvantaged than the elderly living with spouse or living with spouse and adult children. As previous studies proved [12,13], having a spouse is the “greatest guarantee of support in old age” [46]. Marriage can bring health benefits through providing emotional intimacy, economic benefits, social control of behavior and more social integration [47–49], which will in turn influence the health outcome of the elderly. Many studies showed that the elderly living alone were more likely to be depressed than those living with spouses [50–52].

After controlling the confoundings, the tobit regression findings indicated that the living arrangements were associated with HRQOL of the elderly, but these associations differed by regions (urban or rural) and dimensions of HRQOL. In urban area, the single elderly either living alone or living with adult children are disadvantaged than the elderly living with spouse in overall HRQOL, which were consistent with another research exploring the association between living arrangements and HRQOL of the urban elderly in China [24]. However, the regression results in rural area showed that only the single elderly living with adult children had worse health than the elderly living with spouse and another study which used the data from China Health and Retirement Longitudinal Survey(CHARLS) also indicated the same results on the HRQOL of the rural elderly [53]. When we separately analyzed the associations of living arrangements with different dimensions of elders’ health by using the logistic regression models, we found that the urban elderly living alone had worse physical and mental health than the elderly living with spouse, but they had better physical health than the single elderly living with adult children, which was consistent with the results of some previous studies conducted in China [19,20,24,32]. In rural area, the single elderly living with adult children shows the worst physical health, while the mental health of the single elderly living alone was still the worst in the four living-arrangement groups. To conclude all the regression results, we found that the single elderly living with adult children both in urban and rural area had the worst physical health and “living alone” was a significant predictor for the single elderly to report mental problems. The possible reason for the worst physical health of those single elderly who live with adult children might be that living with adult children was a passive choice, for previous studies have shown that disability in Activity of Daily Living (ADL) positively influences the selection of living arrangements for the elderly [19,54]. Elderly with cognitive impairment and ADL disability are more likely to live with family members [20]. If the elderly lost care from their spouses, to live with their children is the only choice for them. In the research of Anderson et al.(1998), it was indicated that the elderly women who live alone appear to be protected against functional declines [55]. However, due to the limitation of our the data, we can’t confirm whether their worse physical health influences their selection of living arrangements or whether coresidence with adult children may negatively influence the health of those single elderly.

Elderly people living alone have been described as an “at risk” group by the World Health Organization [56]. As previous studies has proved that living alone was linked to psychological disadvantages [22,23,50,51], our results also indicated that both urban and rural living-alone elderly reported worse mental health than the elderly living with spouse, but only the urban counterparts showed worse physical health. A prior study which sought to examine whether living alone affects Chinese elders’ physical health and emotional well-being also provided evidence for this phenomenon [57]. We explain these results in several ways. Firstly, based on Chinese traditional values and living preference, most Chinese elderly either in urban or rural area tend to choose to live with their family members when they become single. If they have no family members or the family members are not willing to live with them, and living alone is not their choice, they may feel abandoned, less loved and cared about, and even depressed. Second, although it’s hard to believe that living alone is a physical health protective factor for the rural elderly, due to the less available pension and welfare system in rural area, the rural elderly have to depend on their farmland to make a live and keeping on working on the farm may improve their physical health in return. Therefore, as a result of selective survival, the elderly who live alone might be only those who can physically, emotionally, financially, and socially afford to live alone—they are either healthier or more resourceful than those living with others [57]. In Li’s study, it also pointed that living alone lowers the risk of ADL disability of the elderly [13]. Although the Chinese government had made a law mandating parental visits in 2013, millions of “empty nest” elderly still confronting long-time loneliness and their depression symptoms are very easy to be neglected, compared with their physical health. Combined efforts and support from family, community and governments are needed to improve their psychological well-being.

Almost all the factors listed in this study may influence the HRQOL of the elderly, however, when the five dimensions of EQ-5D were separately analyzed in differentiated regions (urban and rural), those factors also presented different effects on the HRQOL of the elderly. In urban area, the economic condition only influenced the dimension of mobility, but it had effects on all the dimensions of EQ-5D in rural area.Given the poor HRQOL of the rural elderly, it is crucial to strengthen their economic independence. Compared with the female elderly, the male elderly had better HRQOL both in urban and rural area. However, the gender difference in association with five dimensions of HRQOL area was different between urban and rural. In urban area, only the the dimension of pain shows gender difference, but in rural area, the gender difference were significant in regards to all five dimensions in rural area, which indicated that the female elderly in rural area had worse physical and mental health than the male elderly. Policy makers should pay more attention to the female eldlely, especially those living in rural area.

There are some limitations to the current study. First, although the result of the current study may provide some policy implications for governments, our use of the data from Shaanxi province which is loated in the western region of China may limit its applicability to other provinces or the whole country. Second, we aimed to analyze the associations between different living arrangements and HRQOL of older Chinese adults, however, due to the limitation of our household-survey data, we can’t include the elderly living in the institutions and give a whole picture of all the living arrangements. Third, although we have controlled some confoundings, there still be some other potential factors which might cause a deviation of the results, such as dietary habit, smoking or not, disposition of the elderly and so on. Lastly, it is impossible to fully understand the reasons for association between living arrangements with HRQOL of the elderly through quantitative analysis alone and qualitative or mixed methods are needed in future studies to provide us more explanatory insight.

## Conclusions

Living arrangements of the elderly is associated with their HRQOL and there exist heterogeneity in rural and urban areas. Policies that can reduce the health inequity between urban and rural area should seek, for example, to increase financial support and social assistance to the rural elderly. Family support is still the primary and most important support for Chinese elderly, especially the support from a spouse, which plays an important role in maintaining and improving the physical and mental health of the elderly. More attention should be given to those single elderly who are living alone and living with adult children because of the worse mental health of the elderly living alone and worse physical health reported by the single elderly living with adult children both in urban and rural area. In terms of the poor mental health of the living-alone elderly, government, community and family members should work together to build more harmonious surroundings for those elderly and give them more care and attention. Although coresidence is considered as the ideal living arrangement for Chinese elderly, solitary living arrangement doesn’t mean disadvantaged health for all elder adults. For those who can completely live on their own in rural area, living alone may benefit their functional and ADL ability. Additionally, with the increasing number of the oldest old in China, more diversified living options should be provided for the elder adults to choose based on their need and preference.
